# Impact of cold waves on diabetes-related morbidity and mortality: a meta-analysis

**DOI:** 10.3389/fpubh.2025.1696130

**Published:** 2025-12-04

**Authors:** SeoYeon Chung, Jin-Nam Kim, Sungwon Lim, Kyoung-Nam Kim, So-Youn Park, In-Hwan Oh

**Affiliations:** 1 Department of Home Economics Education, School of Human Ecology, Korea University, Seoul, Republic of Korea; 2 Department of Preventive Medicine, Kyung Hee University School of Medicine, Seoul, Republic of Korea; 3 College of Nursing, Dongguk University WISE, Gyeongju, Republic of Korea; 4 Department of Preventive Medicine, Yonsei University College of Medicine, Seoul, Republic of Korea; 5 Department of Hospital Medicine, Inha University Hospital, Inha University School of Medicine, Incheon, Republic of Korea; 6 Department of Preventive Medicine, University of Ulsan College of Medicine, Seoul, Republic of Korea

**Keywords:** environmental exposure, climate change, weather, mortality, morbidity, vulnerability assessment, public health policy

## Abstract

**Introduction:**

Extreme weather events, such as cold waves, pose health risks to individuals with diabetes mellitus. While heat-related health impacts are well-documented, the effects of cold waves remain underexplored. We aimed to review and quantify the health risks associated with cold wave exposure among patients with diabetes.

**Methods:**

A meta-analysis, following Preferred Reporting Items for Systematic Reviews and Meta-Analyses guidelines, was conducted by searching PubMed, SCOPUS, and Web of Science databases from inception up to August 2023. Eight studies met the inclusion criteria for assessing diabetes-related morbidity and mortality risks associated with cold wave exposure. A random-effects model was used to account for heterogeneity.

**Results:**

The meta-analysis revealed a 40% increase in diabetes-related mortality risk (relative risk: 1.40, 95% confidence interval: 1.25–1.58) and a slightly elevated morbidity risk (relative risk: 1.27, 95% confidence interval: 0.99–1.63) during cold waves. Risk variation across studies suggests the influence of environmental and healthcare disparities.

**Conclusion:**

Cold wave exposure significantly increases diabetes-related health risks, highlighting the need for enhanced public health strategies. Further research is needed to address the gaps in understanding cold wave morbidity impacts and related socioeconomic factors.

## 1 Introduction

Changing global climate patterns have precipitated a series of extreme weather events, including heat waves and cold waves, each posing challenges to public health (1). Global warming has been linked to an increase in unusual weather phenomena, notably cold waves, defying climate projections, especially in East Asia and North America, since the 2000s (2). The health consequences of extreme cold have been under-researched despite evidence linking it to mortality risks during winter, especially in developing countries (3). The prolonged impact of cold waves, which can last for weeks, contrasts sharply with the brief, intense effects of heat waves (4). This underscores a significant knowledge gap on the impact of extreme weather conditions on health outcomes, considering the rising global prevalence of chronic diseases, such as diabetes mellitus.

Patients with diabetes mellitus suffer heightened vulnerability to adverse weather conditions. With the increasing prevalence of diabetes worldwide (5), research has begun to focus on how temperature extremes, particularly cold waves, may disproportionately affect individuals with diabetes (6, 7). Most of these studies, however, have primarily focused on the effects of heat and rising ambient temperatures in the context of climate change (6, 8), whereas cold-related risks have received comparatively little attention. This imbalance is striking given that cold waves, as another manifestation of climate variability, tend to persist longer and may impose distinct challenges for diabetes management. Cold weather poses a twofold challenge: it affects physiological aspects of diabetes management, such as insulin sensitivity and glucose metabolism (9), and impacts social determinants of health, such as access to care and disease management resources during adverse weather conditions (10). Consequently, exploring the interplay between cold spells and diabetes management is essential for devising targeted interventions to mitigate these risks and improve health outcomes for this vulnerable group.

While the effects of heat on diabetes have received some attention (6, 11–13), the intricate relationship between cold exposure and diabetes management and outcomes remains underexplored. Preliminary evidence hints at the adverse effects of cold temperature on glucose metabolism, which could exacerbate glycemic control challenges and increase the vulnerability of individuals with diabetes to other cold-induced health risks, such as cardiovascular diseases (14, 15). This meta-analysis aimed to comprehensively evaluate the impact of cold waves on the overall management of diabetes. This study had three objectives: (i) to elucidate the present state of knowledge, (ii) to identify research gaps, and (iii) to propose avenues for future research.

## 2 Methods

### 2.1 Data sources and search strategy

This study was exempted from review by the Institutional Review Board of Kyung Hee University (IRB File No. KHSIRB-23-094) as it did not involve direct human participants or identifiable private information. Consequently, informed consent was not applicable. This systematic review and meta-analysis was retrospectively registered in the International Prospective Register of Systematic Reviews (PROSPERO; Registration No. CRD420251183151, available at https://www.crd.york.ac.uk/PROSPERO/view/CRD420251183151). We conducted a meta-analysis using the Preferred Reporting Items for Systematic Reviews and Meta-Analyses guidelines (16). We searched literature in three major databases—PubMed, SCOPUS, and Web of Science—selected for their extensive coverage of medical and environmental sciences. The search was conducted from database inception to August 31, 2023, to include the most recent studies. The search strategy was designed to encompass a broad range of studies by using a combination of keywords and phrases related to cold exposure and diabetes. The specific search terms employed were: (“cold spell” OR “cold related” OR “cold weather” OR “cold temperature”) AND (diabetes OR “diabetes mellitus”).

### 2.2 Study selection

Studies underwent a rigorous screening process based on pre-defined inclusion and exclusion criteria. The inclusion criteria were: (1) studies evaluating the impact of cold waves on diabetes morbidity or mortality; (2) definition of diabetes mellitus according to the International Classification of Diseases-9 code 250 or International Classification of Diseases-10 codes E10–E14; (3) availability of relative risk (RR) and 95% confidence intervals (CIs) as outcome measures; and (4) publications in English. The exclusion criteria encompassed studies focusing solely on emergency room visits, cardiovascular mortality in patients with diabetes, and seasonal variations in blood sugar levels without direct attribution to cold wave events.

### 2.3 Meta-analysis

The meta-analysis evaluated the RR of health effects associated with diabetes due to cold wave exposure. Definitions of cold waves varied across studies, with some applying absolute temperature thresholds and others using percentile-based criteria with different duration requirements. Specifically, several studies defined a cold wave as a period when the daily mean temperature remained below the 5th or 10th percentile of the local temperature distribution for at least two to four consecutive days (3, 17). Others adopted definitions based on national meteorological standards, such as those of the Korea Meteorological Administration, which issues cold-wave advisories when the morning minimum temperature falls below −12 °C and warnings when it falls below −15 °C for two or more consecutive days. Among these studies, seven examined mortality, while two investigated morbidity as health outcomes. Due to the inherent variability in study designs, populations, and contexts, a random-effects model was used, accommodating the expected statistical heterogeneity among the studies. Heterogeneity was assessed using the Q test, τ^2^, and the I^2^ statistic, which quantifies the percentage of total variation across studies attributable to heterogeneity rather than chance. For example, in the mortality analysis, heterogeneity was moderate (I^2^ = 63.9%, Q[df = 10] = 27.7, *p* < 0.01, τ^2^ = 0). Given these results, the use of a random-effects model was appropriate. Study-level characteristics were also considered when interpreting sources of heterogeneity.

## 3 Results

The literature search yielded a total of 372 articles. After excluding duplicates, 304 remained. From this pool, eight studies were selected based on their direct relevance to the research question and adherence to the predefined inclusion criteria (Table 1). The eight studies included in this analysis spanned a wide geographical range, encompassing one study from Korea, four from China, one from Hong Kong, one from the Philippines, and one from Canada.

**Table 1:** Articles included in the meta-analysis of cold wave exposure on diabetes-related health outcomes.

Study	Year of publication	Country	Study duration	Population	Diabetes-related morbidity or mortality	Area	ICD-10 code	Data source	Model	Definition of cold exposures	RR	95% CI	Additional information
Bai et al. (17)	2016	Canada	1996–2013	14,000,000	Morbidity	Province of Ontario	E10–E14	Hospital admissions data from Canadian Institute for Health Information	DLNM combined with quasi-Poisson regression	Comparison between cold threshold and reference value (1st percentile vs. minimum morbidity temperature percentile)	1.12	1.01, 1.24	
Kim et al. (7)	2022	Republic of Korea	2010–2019	51,318,454	Morbidity	Overall	E10–E14	Hospital admissions data from National Health Insurance Service	DLNM combined with quasi-Poisson regression	Temperatures below cold threshold compared with temperatures above threshold (below vs. above 5th percentiles)	1.45	1.26, 1.66	
					Mortality	Overall	E10–E14	Cause-specific mortality data from Statistics Korea			2.02	1.37, 2.99	
Ma et al. (20)	2020	China	2014–2017	72,500,000	Mortality	11 cities in Jiangsu Province	E10–E14	Cause-specific mortality data from Jiangsu Provincial Center for Disease Control and Prevention	DLNM combined with quasi-Poisson regression	Comparison between cold threshold and reference value (2.5th percentile vs. minimum mortality temperature percentile)	1.24	0.84, 1.82	
Liu et al. (21)	2020	Hong Kong	2006–2016	-	Mortality	Overall	E10–E14	Mortality data from Hong Kong Census and Statistics Department	DLNM combined with quasi-Poisson regression	Comparison between cold threshold and reference value (1st (10th) percentile vs. minimum mortality temperature percentile)	1.58	0.84, 2.98	Extreme cold
											1.34	0.88, 2.03	Cold
Su et al. (19)	2020	China	2014–2017	141,820,000	Mortality	17 cities	E10–E14	Mortality data from local Centers for Disease Control and Prevention	DLNM combined with quasi-Poisson regression	Comparison between cold threshold and reference value (2.5th percentile vs. minimum mortality temperature percentile)	0.95	0.50, 1.81	
Chen et al. (3)	2019	China	2007–2013	-	Mortality	31 capital cities	E10–E14	Mortality data from Chinese National Center for Chronic and Non-communicable Disease Control and Prevention	DLNM combined with quasi-Poisson regression	Temperatures below and above cold threshold compared (below vs. above 5th percentiles)	1.48	1.30, 1.66	Lag 0–14
											1.66	1.43, 1.89	Lag 0–27
Seposo et al. (22)	2017	Philippines	2006–2011	7,272,000	Mortality	4 cities	E10–E14	Cause mortality data from Philippine National Statistics	DLNM combined with quasi-Poisson regression	Comparison between cold threshold and reference value (1st (10th) vs. 25th percentiles)	1.20	0.54, 2.69	Extreme cold
											1.10	0.71, 1.71	Cold
Yang et al. (18)	2016	China	2007–2013	129,300,000	Mortality	9 large cities (Beijing, Shanghai, Shenyang, Harbin, Chengdu, Chongqing, Kun-ming, Changsha, and Guangzhou)	E10–E14	Chinese National Center for Chronic and Noncommunicable Disease Control and Prevention	DLNM combined with quasi-Poisson regression	Comparison between cold threshold and reference value (1st (10th) vs. 25th percentiles)	1.44	1.25, 1.66	Extreme cold
											1.20	1.12, 1.30	Cold

DLNM, distributed lag non-linear model; ICD, International Classification of Diseases.

### 3.1 Mortality and morbidity risk associated with cold exposure

Studies from various countries have investigated the relationship between cold exposure and diabetes-related health outcomes, revealing a mix of results that highlight regional and methodological differences.

In terms of morbidity findings, a study conducted by Bai et al. from 1996 to 2013 with a sample size of 14 million in Ontario, Canada, examined the risk of diabetes-related morbidity during cold weather (17). Using hospital admissions data and applying a distributed lag non-linear model with quasi-Poisson regression, the study obtained an RR of 1.12 (95% CI: 1.01–1.24) for morbidity due to cold exposure. This suggests a statistically significant, albeit moderate, increase in the risk of diabetes-related hospital admissions associated with cold weather conditions. Furthermore, studies from China also supported a significant morbidity risk. Chen et al. focused on the broader Chinese population from 2007 to 2013 (3). The study found a significant morbidity risk associated with cold exposure, with an RR of 1.48 (95% CI: 1.32–1.66) for temperatures below the cold threshold, using a distributed lag model for analysis. Yang et al. analyzed both morbidity and mortality in China from 2007 to 2013 (18). Using a distributed lag non-linear model, the study reported an increased risk of morbidity with an RR of 1.44 (95% CI: 1.25–1.66) when comparing cold threshold temperature with a reference, highlighting significant risk associated with cold exposure. A study from South Korea by Kim et al. extended this research by analyzing the entire national population of 51,318,454 from 2010 to 2019 (7). Using a distributed linear lag model combined with a generalized additive model, the study reported a higher RR of 1.45 (95% CI: 1.27–1.66) for diabetes-related morbidity due to cold exposure. This finding suggests a more pronounced risk increase in colder climates, emphasizing the significant impact of cold weather on the health of individuals with diabetes.

In terms of mortality findings, several studies investigated the relationships between cold exposure and diabetes-related mortality. Yang et al. (2007–2013) found a significant association between cold exposure and increased mortality in China. This study identified a significant association between cold exposure and diabetes-related mortality (RR: 1.20, 95% CI: 1.11–1.30), underscoring the severe impact of cold weather on the survival rate of patients with diabetes. Su et al. analyzed the impact of cold exposure in 14 cities across China from 2014 to 2017 (19). The mortality risk related to cold exposure had an RR of 0.95 (95% CI: 0.50–1.81), indicating no significant risk increase. In contrast, Ma et al. studied the mortality effects of cold exposure in 11 cities in Jiangsu Province, China, from 2014 to 2017 (20). Data from multiple cities with a combined population of 72,500,000 were analyzed using a distributed lag non-linear model. Cold exposure was defined as temperature below the cold threshold compared to a reference. They reported an RR of 1.24 (95% CI: 0.84–1.82), indicating a non-significant increase in mortality risk. In Hong Kong, Liu et al. conducted a study over a decade from 2006 to 2016 (21), highlighting the mortality risk associated with cold exposure, which was defined similarly using a temperature threshold comparison. The reported RR was 1.34 (95% CI: 0.88–2.03), suggesting a trend toward increased mortality risk, although the CI suggests uncertainty. Finally, Seposo et al. extended the geographical scope to the Philippines, covering the period from 2006 to 2011 (22). Despite the tropical climate, the study reported an RR of 1.20 (95% CI: 0.54–2.69) for diabetes-related mortality, indicating a potential risk increase due to cold spells.

### 3.2 Mortality risk

**Figure 1:**
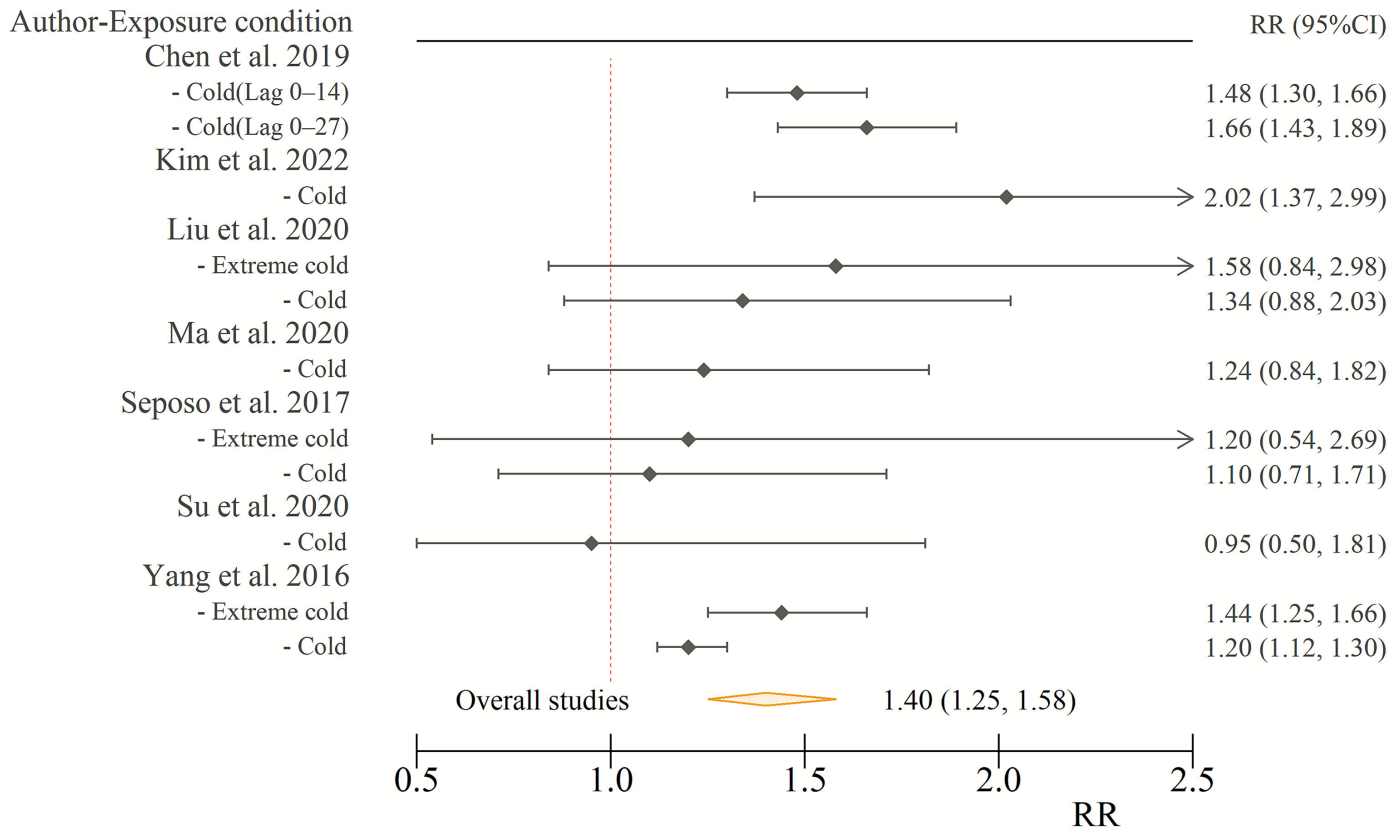
Relative risk of diabetes-related mortality associated with cold wave exposure. CI, confidence interval; RR, relative risk.

The meta-analysis revealed a significant association between cold wave exposure and increased mortality risk in individuals with diabetes. The pooled RR of diabetes-associated mortality due to cold wave exposure was 1.40 (95% CI: 1.25–1.58). This represents a 40% increase in the risk of mortality for individuals with diabetes during cold wave conditions compared to non-cold wave conditions (Figure 1).

### 3.3 Morbidity risk

**Figure 2:**
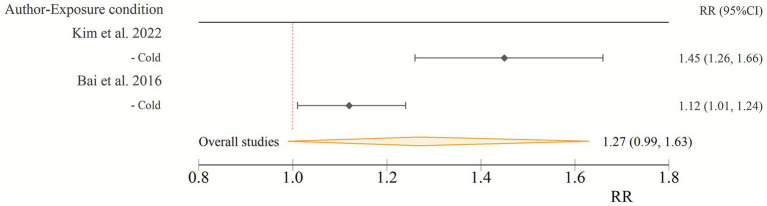
Relative risk of morbidity in patients with diabetes due to cold wave conditions. CI, confidence interval; RR, relative risk.

Similarly, an elevated risk of diabetes-related morbidity was observed, albeit with a wider CI. The pooled RR for morbidity in patients with diabetes due to cold wave conditions was 1.27 (95% CI: 0.99–1.63). Despite the lower bound of the confidence interval being marginally below unity, this result suggests a notable increase in the risk of morbidity due to diabetes during cold wave exposures (Figure 2).

## 4 Discussion

This meta-analysis shows that extreme cold increases the risk of health complications and mortality among people with diabetes. The risk was particularly elevated for mortality, but the magnitude and significance differed across populations and regions.

Most prior studies have emphasized the health impacts of heat exposure. For example, Zanobetti and Schwartz reported higher hospital admissions and mortality among diabetes patients during heatwaves (23). Basu (24) and Semenza et al. (25) also linked heat exposure to increased blood glucose, cardiovascular events, and mortality. Experimental studies also provide mechanistic evidence; for instance, extreme heat combined with traffic-related air pollution (NO₂) has been shown to disrupt metabolic and autophagy pathways in a type II diabetes mouse model, thereby contributing to diabetic nephropathy (26). Comparable mechanisms—including sympathetic activation, elevated blood pressure, impaired insulin sensitivity, oxidative stress, and inflammatory responses—have also been described for cold exposure (9, 27). Taken together, these mechanisms suggest that cold waves, much like heat exposure, may pose substantial risks of morbidity and mortality in diabetes, supporting the rationale for our analysis.

Our findings strengthen the evidence that cold exposure contributes to diabetes-related mortality. Yang et al. analyzed nine Chinese cities (2007–2013) and reported higher risks on extremely cold days, with diabetes mortality 44% higher during extreme cold and 20% higher during less severe cold (18). Chen et al. extended this evidence by examining 31 cities during the same period. They found that cold spells increased mortality with RRs of 1.48 (95% CI: 1.30–1.66) over lag 0–14 days and 1.66 (95% CI: 1.43–1.89) over lag 0–27 days (3). These findings are consistent with our analysis, confirming a significant association between cold wave exposure and diabetes-related mortality. They also suggest that contextual factors, such as healthcare access and socioeconomic disparities, should be considered when interpreting regional differences in risk.

However, not all studies reported consistent findings. Li et al. examined two Chinese cities and observed contrasting results. Notably, these cities differ markedly in their local climates. In Harbin, each 1 °C decrease from −11 °C in daily maximum temperature was associated with a 13% higher mortality risk (RR = 1.13, 95% CI: 1.03–1.34). In contrast, in Chongqing, the estimated effect was similar but not statistically significant (RR = 1.13, 95% CI: 0.95–1.48) (28). This difference suggests that residents in Harbin, where the average winter temperature is −14.6 °C under a cold continental climate, may experience greater adverse health effects from extreme cold, whereas those in Chongqing, which has a humid subtropical climate with mild winters averaging 7.8 °C, may have higher physiological and behavioral adaptation to such conditions. Liu et al. analyzed mortality in Hong Kong from 2006 to 2016 and reported elevated risks, with an RR of 1.58 (95% CI: 0.84–2.09) for the coldest days and 1.34 (95% CI: 0.88–2.03) for moderately cold days, though these associations did not reach statistical significance (29).

Similarly, Ma et al. (20), conducted in Jiangsu Province within a temperate climate, reported a higher relative risk than Su et al. (19), which analyzed 17 cities spanning both temperate and subtropical regions. This discrepancy may be partly related to differences in local climatic conditions. These findings suggest that in relatively warm subtropical regions, the health effects of cold spells may be less pronounced, highlighting the need for further research. Beyond climatic differences, populations with better healthcare access, higher socioeconomic status, and greater public awareness may be better protected against cold-related complications, whereas rural or disadvantaged communities often face higher risks (30, 31). These patterns underscore the importance of developing region- and climate-specific interventions to mitigate the health impacts of cold exposure among people with diabetes.

Previous studies also support the findings that contextual factors influence vulnerability to cold-related health risks. For instance, Analitis et al. showed that mean winter temperature modified cold-related mortality across 15 European cities, with stronger effects observed in warmer areas (32). Healy found that excess winter mortality in Europe was closely linked to poverty and deprivation (33). In the United States, O’Neill et al. reported that racial minorities, lower education groups, and deaths outside hospitals were associated with higher vulnerability (34). Wilkinson et al. similarly found that older adults with preexisting illness in Great Britain were disproportionately affected (35). Together, these findings show that differences in healthcare access, adaptive capacity, and social conditions explain much of the heterogeneity observed across regions. Our results should therefore be interpreted in light of these broader contextual factors.

Most research on cold exposure in diabetes has focused on mortality, with morbidity receiving far less attention. Hajat et al. (36) and Song et al. (37) noted this gap, while Medina-Ramón et al. (38) and Schwartz (39) suggested that cold exposure may worsen outcomes among individuals with chronic conditions, including diabetes. Few studies, however, have directly investigated diabetes morbidity during cold spells. By examining hospital admissions as indicators of disease exacerbation, our study adds complementary evidence, in line with prior findings of greater cardiorespiratory risks during cold seasons in people with diabetes and prediabetes (40). By considering both mortality and morbidity, our study provides a clearer understanding of the health impacts of cold exposure in diabetes. While both outcomes indicated elevated risks, the morbidity findings should be interpreted with caution, given the wider confidence intervals and the limited number of available studies.

This study has some limitations. First, we could not control for co-existing morbidities among patients with diabetes, such as renal and heart diseases, which may be influenced by extreme temperatures and contribute to increased mortality. Second, the absence of individual-level data prevented assessment of socioeconomic factors, such as marital status and household income, which could act as confounders. Third, the funnel plot indicated a potential publication bias, which may have led to an overestimation of the pooled effects; therefore, the findings should be interpreted with caution. Fourth, the lack of a standardized definition for a cold wave across the included studies is a notable limitation. Included studies used diverse criteria, including absolute temperature thresholds and percentile-based definitions with different duration requirements. This methodological inconsistency likely contributed to the high degree of heterogeneity observed in our results and may limit the direct comparability of findings across studies. Accordingly, the certainty of evidence for some outcomes should be interpreted as limited, primarily due to inconsistency across studies and imprecision related to wide confidence intervals and the small number of available studies. Despite these limitations, our study has several notable strengths. Most importantly, it is one of the first to examine cold wave–related risks in diabetes by addressing both morbidity and mortality outcomes together, an area largely overlooked in prior research. In addition, by drawing on evidence from diverse geographic settings and large-scale data, the study reflects a wide range of climatic and healthcare contexts, highlighting the need for policies that address these contextual differences. These features underscore the need to integrate climate adaptation into chronic disease management and highlight policy implications for reducing cold-related risks in diabetes.

In conclusion, cold wave exposure was associated with an increased risk of diabetes mortality (RR = 1.40, 95% CI: 1.25–1.58) and a clear rise in morbidity. These findings underscore the vulnerability of people with diabetes, particularly as cold waves are expected to become more frequent and intense under climate change. The heterogeneity across regions points to the role of environmental, socioeconomic, and healthcare factors in shaping risks. Future research should examine these determinants alongside biological mechanisms such as insulin sensitivity and glucose metabolism. Longitudinal and multidisciplinary studies are needed to clarify how climate conditions and healthcare systems interact to influence outcomes. This study provides a basis for developing targeted public health strategies to protect people with diabetes during cold waves. Linking weather alert systems with diabetes registries, enhancing community-based monitoring of high-risk patients during cold spells, and ensuring uninterrupted access to essential medications and supplies could help reduce cold-related risks and promote health equity in this vulnerable population.

## Data Availability

The original contributions presented in the study are included in the article/supplementary material, further inquiries can be directed to the corresponding author.
